# Supplementary material for: The Construction and Exploration of a Comprehensive MicroRNA Centered Regulatory Network in Foxtail Millet (Setaria italica L.)
Source: Front Plant Sci. 2022 May 6;13:848474. doi: 10.3389/fpls.2022.848474 (PMC9121102; doi:10.3389/fpls.2022.848474)
Supplement: Supplementary file 3 [file Data_Sheet_3.zip › Sit-miR396a_Seita.3G138800.1_375_TPlot.pdf]

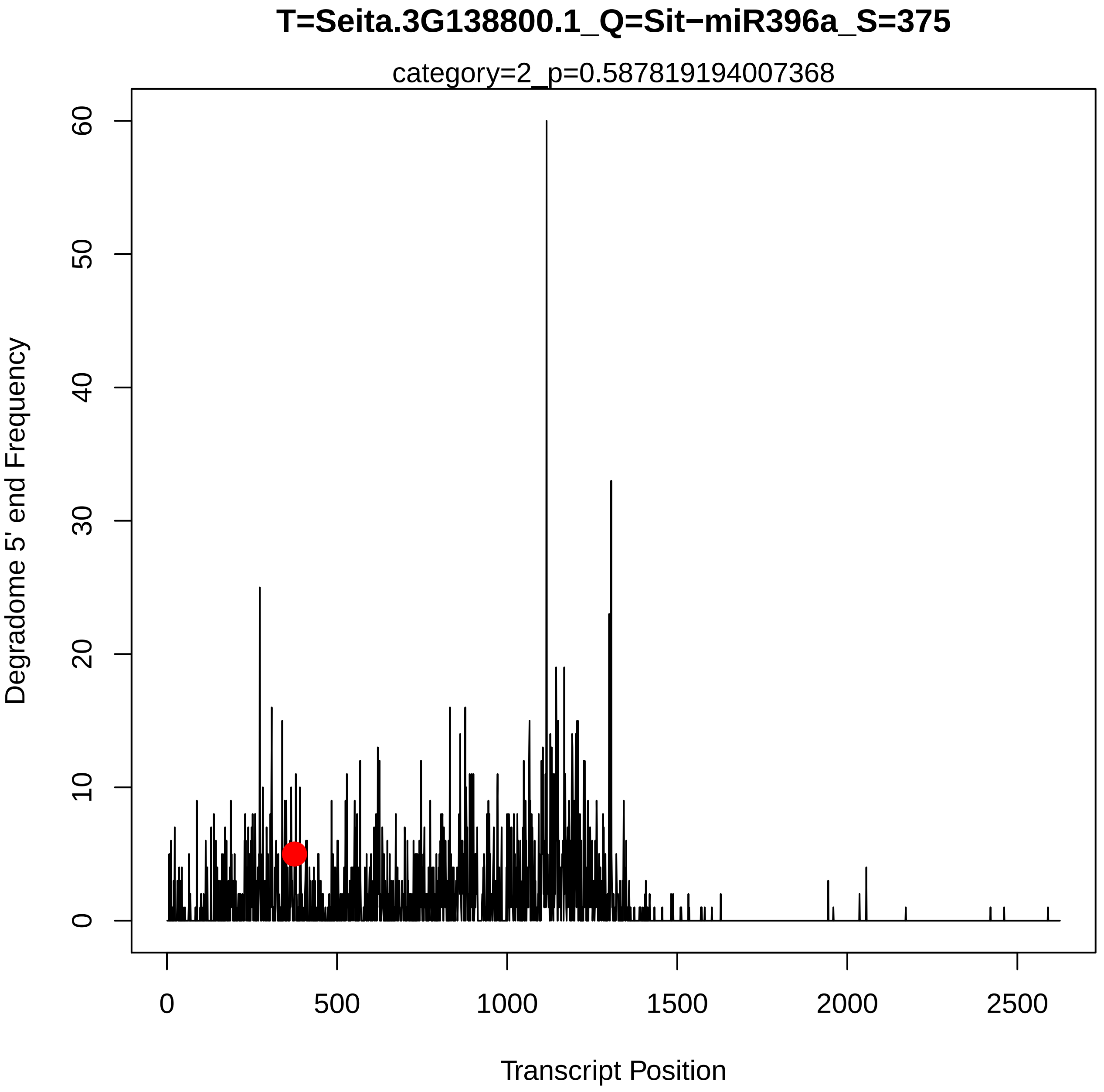
T=Seita.3G138800.1_Q=Sit−miR396a_S=375
category=2_p=0.587819194007368
Degradome 5' end Frequency
0
10
20
30
40
50
60
Transcript Position
0
500
1000
1500
2000
2500
